# Memory and Entropy

**DOI:** 10.3390/e24081022

**Published:** 2022-07-24

**Authors:** Carlo Rovelli

**Affiliations:** 1 Aix Marseille University, Université de Toulon, CNRS, CPT, 13288 Marseille, France; rovelli.carlo@gmail.com; 2 Perimeter Institute, 31 Caroline Street North, Waterloo, ON N2L 2Y5, Canada; 3 The Rotman Institute of Philosophy, 1151 Richmond St. N, London, ON N6A 5B7, Canada

**Keywords:** traces, metastable systems, information

## Abstract

I study the physical nature of traces. Surprisingly, (i) systems separation with (ii) temperature differences and (iii) long thermalization times are sufficient conditions to produce macroscopic traces. Traces of the past are ubiquitous because these conditions are largely satisfied in our universe. I quantify these thermodynamical conditions for memory and derive an expression for the maximum amount of information stored in such memories as a function of the relevant thermodynamical parameters. This mechanism transforms low entropy into available information. I suggest that all macroscopic information has this origin in past low entropy.

## 1. The Problem

The present abounds with traces of the past (footsteps in the sand, craters on the moon, geological strata, photos of us younger, etc.) without analogous traces of the future. We remember the past, not the future, and this might be the very source of the psychological and the epistemic arrows of time [1,2]. What is the physical mechanism giving rise to this time asymmetry and why there is such a great abundance of traces of the past in nature?

The second law of thermodynamics is the only “fundamental” law (including in quantum physics [3,4]) that breaks time-reversal invariance; hence, traces must be macroscopic phenomena related to an entropy gradient and ultimately to past low entropy [5]. Why does past low-entropy yield the ubiquity of traces of the past, and how?

Building on [5,6], I show here that the combined presence of (i) systems separation; (ii) past low entropy, in the form of a temperature difference between systems; and (iii) long thermalization times is a sufficient condition to produce traces of the past.

Using this result, I derive an expression for the maximum amount of information stored in memory in this way as a function of the relevant thermodynamical parameters.

The problem discussed here is not how to characterize the physical meaning of “traces” in general. It is to understand why there are so many traces of the past around us. More precisely, what is the general thermodynamical mechanism—which appears to be in place in the universe—that generates the abundance of traces we see?

The relationship between memory and the second law is studied from a different perspective also in [1].

A warning about terminology. Following the habit of physicists, I use the expressions “memory” and “trace” interchangeably (a rock “keeps the memory” of the ancient volcanic activity). The notion of memory that we use in everyday life is something similar to a psychological state representing past events. Here, I do not address at all the specific aspects of “memory” in this psychological sense.

## 2. A Concrete Model for Memory

I use the expressions “memory” or “trace” as synonyms to indicate a feature of the present configuration of the world that we promptly identify as witnessing a specific past event. Examples are photos, written texts, footsteps, impact craters, memories in brains and computers, fossils, gravitational waves emitted from a black hole merger, this very article you are reading, and so on. There are no analogous traces of future events, in our experience. To pinpoint the nature of these memories, I describe a simple paradigmatic model.

Consider two physical systems that interact occasionally. The first, say, is formed by balls moving freely in a closed room, bouncing elastically on the walls and on each other. Assume that the balls are sufficiently heavy and elastic to make any dissipation negligible and that have been bouncing long enough to average out their energy. The second is formed by several pendulums hanging on ropes from the ceiling. For these, assume that friction in the rope and with air is non-negligible. Hence, oscillations of the pendulums are damped. The two systems interact when one of the balls happens to hit elastically one of the pendulums. Assume that conditions are such that collisions between the balls and the pendulums happen, but are rare, during the time span considered.

**Figure 1:**
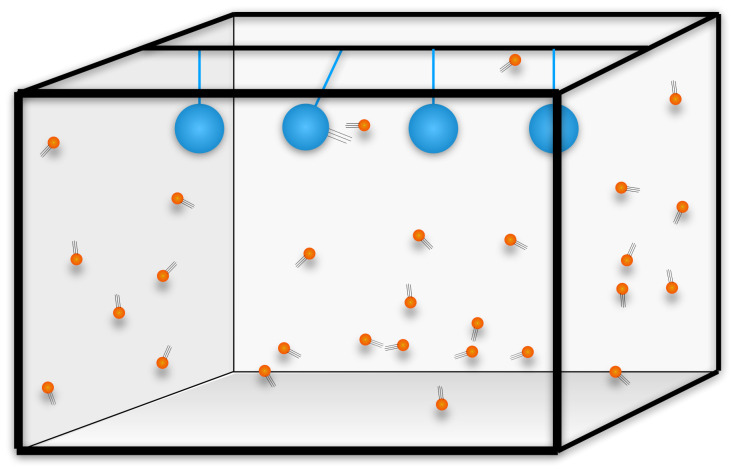
The model for memory. The cold damped pendulums and fast hot balls. The wide oscillation of a single oscillating pendulum is a trace of a past interaction between a ball and a pendulum.

Call “event” a collision between a ball and a pendulum. Consider histories where the pendulums are initially near rest, undergoing small thermal fluctuations, while the balls move fast. Anytime a ball hits a pendulum, it sets it in motion, and the pendulum oscillates for a while. Its oscillations are slowly damped by friction. Consider the state of this system at some time *t*. See Figure 1. Say that we see most pendulums near rest, except for a few that oscillate widely. From these oscillations, we infer that those pendulums were hit by a ball *in the past*. This is an example of “trace“, or “memory”. The memory, namely, the oscillation, lasts for a while. Long after the hit, the excited pendulums are back to near rest, as they were initially. The state of the pendulums at time *t* has information about events (interactions between balls and pendulums) in the past, but not in the future. Something has broken time-reversal invariance. What, and how?

The first point to observe is that the description above is macroscopic. By this, I mean that the only relevant information given is that “the balls move fast” and “the pendulums are near rest”. This is *averaged* information.

A trace is an irreversible phenomenon. This can be seen here by running the history backward in time: one of the few wildly oscillating pendulums collides with a ball that happens to absorb its energy nearly entirely. This looks very implausible, namely, highly improbable. If the phenomenon is irreversible, it needs an initial low entropy, to break time-reversal invariance. Where is the initial low entropy?

The answer is that the initial low entropy is in the temperature difference between the two systems (balls and pendulums) at the past end of the time interval considered. This, in fact, decreases slowly at each event. Indeed, let $E_{e}$ (“*e*” for environment) be the average energy per degree of freedom of the balls. This defines a temperature $T_{e}$ via $E_{e}=\frac{1}{2}kT_{e}$. Here, *k* is the Boltzmann constant. Let $E_{m}$ (“*m*” for memory) be the average energy per degree of freedom of the pendulums. This defines a temperature $T_{m}$ via $E_{m}=\frac{1}{2}kT_{m}$. If the two systems had the same temperature—namely, if $T_{e}=T_{m}$—all degrees of freedom would have the same average kinetic energy, the transfer of energy at each ball–pendulum collision would be equally probable from the balls to the pendulums or vice versa, and there would be no memory.

To have memory, we need the balls to have more average energy than the pendulums—that is, we need $T_{e}>T_{m}$. The initial difference of temperature is needed here to have memory. This difference of temperature is a past low-entropy condition, because maximal entropy requires $T_{e}=T_{m}$.

At each collision, the entropy grows because part of the energy is passed from the balls to the pendulums and the temperature difference decreases. If a small amount of energy ΔE is transmitted to a pendulum in a collision, this gives rise (in due time after the energy of the pendulum is dissipated) to an entropy increase,

(1) $$\Delta S∼\frac{\Delta E}{T_{m}}-\frac{\Delta E}{T_{e}}.$$

because in thermodynamical terms, the transferred energy here behaves similar to heat transferred. In a time-reversed history, a pendulum decreasing its energy by transmitting it to a ball is precisely similar to heat going from a cold to a hot body, violating the second law because it is improbable, although mechanically possible.

To have memory, we also need $T_{e}>T_{m}$ to persist, namely, we need the interactions between balls and pendulums to be sparse. Thus, we need the coupling between the two systems to be sufficiently weak to hold them from converging too rapidly to equilibrium. That is, the thermalization time $\tau_{em}$ of the coupled system must be long on the scale of the observation time $t_{tot}$.

We also need the damping of the oscillations of the pendulums to be sufficiently slow to hold the memory. That is, the thermalization time $\tau_{m}$ for the pendulum systems must be sufficiently long on the scale of the time $t_{m}$ we want the memory to last. In other words, we need the pendulums themselves to be sufficiently weakly coupled to hold them from converging rapidly to equilibrium. The memory, or trace, is then neatly identified as a configuration (of a pendulum) that is out of equilibrium (with rope, air, and other pendulums) and remains so for a while thanks to the long thermalization time $\tau_{m}$.

Notice that the initial temperature difference breaks time-reversal invariance. The set of dynamical histories compatible with it is much smaller than the set of histories compatible with the smaller temperature difference at a later time.

## 3. The Nature of Memory

The model discussed above points to a set of simple ingredients sufficient to lead to traces:

(a) System separation. In the example: the balls and the pendulums. Similarly, the meteorites population and the moon’s surface, men walking and the sand, and people and the film of the camera. The separation must be sufficient to permit that thermalization is avoided for a long time lapse with respect to the memory time. That is, the thermalization time $\tau_{em}$ of the composite system must be much longer than the time lapse considered $t_{tot}$.
(b) Past thermodynamical imbalance, for example, a temperature difference. For instance, there should be higher average energy (hence, temperature $T_{e}$) in one of the two systems (balls, meteorites, walking people, colliding black holes) than in the other $T_{m}$. Denote “environment” as the systems with higher temperature and “memory” as the systems with lower temperature.
(c) Long thermalization time in the memory system. The pendulums should not thermalize too fast for memory to hold. The sand should not be equalized by the wind too fast, the photo should not fade too fast, the gravitational waves should not sink in the background radiation, etc. That is, the thermalization time of the memory system $\tau_{m}$ must be longer than the expected duration $t_{m}$ of the memory.

These ingredients are sufficient to give rise to the phenomena that we recognize as traces, or memories, without any need of anything else. Traces are the temporary (but long lasting, because of $\tau_{m})$, out of equilibrium configurations of the “memory” system that *follow* the occasional interactions between environment and memory. They are in the *future* of the interaction because of the time orientation sourced by the initial low entropy.

That is, in general, let us have two systems that we call environment and memory at temperatures $T_{e}$ and $T_{m}$, respectively. Let $\tau_{em}$ be the thermalization time for the coupled system environment+memory and $\tau_{m}$ be the thermalization time in the memory system alone. For a total time lapse $t_{tot}$, we have memories lasting a time of order $t_{m}$ if

(a) $\tau_{em}>t_{tot}$,
(b) $T_{e}>T_{m}$,
(c) $\tau_{m}>t_{m}$.

It is remarkable that such simple conditions are sufficient to generate traces.

These conditions are obviously largely realized in our universe, where many subsystems interact weakly and thermalization is often very slow, giving rise to very common metastable states.

The scheme described here can be generalized, for instance, to cover chemical potential or pressure disequilibria, but it seems to me to capture the core of the reason for the abundance of traces in the universe and their time orientation.

In the next section, we consider a few examples that illustrate this point.

## 4. Examples

•*A stone falls in a quiet pond.* The impact of the stone on the pond generates outgoing concentric waves centered at the location of the impact. These can be viewed as traces, preserving the information about the impact that happened *in the past*, and its location. The key point here is that the water was initially near equilibrium; the molecules had an average kinetic energy ϵ determined by the temperature $T_{m}$ of the pond, where ϵ∼kT. The in-falling stone, on the other hand, has an energy *E* that is higher than ϵ. It is the inequality E≫ϵ—*hence, a thermodynamical reason*—that determines the positive sign of the amount of energy ΔE transferred from the stone to the water. The impact generates outgoing concentric waves (that carry energy) only if E≫ϵ. If the stone had been at thermal equilibrium with the pond, we would have had E∼ϵ (by equipartition of energy) and no outgoing concentric waves would form: the impact between the stone and the water would produce effects indistinguishable from normal thermal motion in the pond. No macroscopic trace of the impact would form.

After the impact, the outgoing concentric waves last only for a finite amount of time because their energy dissipates into the heat of the water. Therefore, we recognize the general thermodynamical structure described above: in the past, there is no equilibrium, the “effective temperature” $T_{e}=E/k$ of the stone is higher than the temperature $T_{m}$ of the water; hence, something in the past had prevented the thermalization of stone and water. In other words, $\tau_{em}$ is larger than the observation time. The time taken by the waves to dissipate can be identified with $\tau_{m}$. Long after this time, the trace has dissipated. The increase of entropy associated to the formation of the trace is $S=\delta E/T_{m}$.

•*A crater on the moon* has exactly the same thermodynamic structure as the concentric waves produced by the stone falling in a stone. The difference is that $\tau_{m}$ is much longer: it is the time erosion that dissipates the crater.

•*A photographic plate* exemplifies a slightly richer mechanism for trace formation. The energy of a photon leaving a mark on the plate may be negligible. However, the grains of the plate are in a metastable state, protected by a thermalization time $\tau_{e}$ much larger than the relevant observation time. The photon triggers a transition of some of the plate’s grains from a lower entropy to a higher entropy state, generating entropy and, hence, leaving a localized mark (a trace) that keeps memory about the landing of the photon and its location. Again, this is protected by dissipation by a long enough $\tau_{m}$.

•*Measurement apparatus* are physical systems *M* that generate and conserve records about quantities of another system *S*. A record means we expect that after the measurement some pointer variable of *M* is correlated with some property of the system *S*. This cannot be realized without dissipation [6] for the following reason. Since microphysics is invariant under time reversal, arbitrary data can be given at any time; therefore, there are always mechanical histories that solve the equations of motion such that after the measurement the correlation is not realized. Why do we discard this possibility in real life? It is because these histories are thermodynamically suppressed. Hence, there is no measurement without dissipation, and thus, entropy production.

• Finally, *computer or brain memories* are obviously largely dissipative processes.

## 5. Information in Memory

Let the heat capacity of the memory system be $C_{m}$, and let us take the heat capacity of the environment to be infinite for simplicity (namely, let us assume that the environment is large). Then, as time passes, the temperature of the memory rises. This can be simply modeled over long enough time scales as

(2) $$T_{m}\left(t\right)=T_{e}-\left(T_{e}-T_{m}\right)e^{-t/\tau_{em}}.$$

Individual memories are lost after a time $∼\;\tau_{m}$. The total energy transmitted during a time lapse $\tau_{m}$ is of the order

(3) $$\Delta E=C_{m}\;\left(T_{m}\left(\tau_{m}\right)-T_{m}\right).$$

This is, therefore, the average energy dropped into the memory system and not yet thermalized, at any given time. With (2), this gives

(4) $$\Delta E=C_{m}\left(T_{e}-T_{m}\right)\left(1-e^{-\tau_{m}/\tau_{em}}\right).$$

Using (1), this energy gives rise to an entropy change

(5) $$\Delta S=\frac{C_{m}{\left(T_{e}-T_{m}\right)}^{2}}{T_{e}T_{m}}\left(1-e^{-\tau_{m}/\tau_{em}}\right).$$

This is the entropy increase from which the information stored in the memory is sourced, under the conditions given.

Memory contains information. This information must come from somewhere. The only possible source is the entropy increase, since entropy increase is information loss. Therefore, (5) determines the maximal amount of information *I* that can be stored in memory in this way:

(6) $$I<\Delta S/k=\frac{C_{m}{\left(T_{e}-T_{m}\right)}^{2}}{kT_{e}T_{m}}\left(1-e^{-\tau_{m}/\tau_{em}}\right).$$

Notice that this is a mechanism that transforms initial low entropy (free energy) into available information.

More precisely, a *macroscopic state* (in the present) has information, in the sense of Shannon’s relative information, about a macroscopic event in the past: the first implies the second. This correlation, however, is not implied by the mechanical laws; rather, it is vouched statistically. How does such (Shannon’s relative) information come about? The answer is that it was sourced by the initial low entropy. Low entropy is information, stored in the relative rarity of microstates. The formation of traces is a mechanism that generates entropy, namely, it consumes this information and transforms (part of) it into macroscopic information.

This transformation of past low entropy into available information plays a role in phenomena such as the biosphere, where available information plays a huge role.

In our universe, system separation and very long thermalization times ($\tau_{em}$) are very common. By far, the dominant one is the lack or thermodynamical equilibrium between helium and hydrogen. Helium and hydrogen ceased to be in thermal equilibrium since nucleosynthesis, because the rapid cosmological expansion (matter was out of equilibrium with the scale factor [7]) lowered the temperature to a point where the thermalization time became much longer than the cosmological times. Helium and hydrogen have remained out of equilibrium since and this disequilibrium is currently the main source of free energy in the universe [7]. Occasionally, energy is dumped from hydrogen to helium: this happens when a star forms and burns. These events are fueling all the free energy that nurtures the biosphere. They are a perfect realization of the mechanism described here and they, indeed, leave very abundant traces of themselves and of their products.

I have long being puzzled by how a disorganized universe, where in the far past matter was in thermal equilibrium, could have spontaneously evolved into the abundance of informative traces of the past that we see around us. It seems to me that the mechanism described here provides the answer.

Furthermore, it identifies the physical source of the entire amount of information in biology, culture, data, books, and so on: it is the past low entropy of the universe.

The idea initially suggested in this work was later developed in the article [8] and more broadly in [9].

## Data Availability

Not applicable.
